# Gait, Quality of Life, and Knee Function in Advanced Knee Osteoarthritis: A Single-Center, Prospective, Observational Study

**DOI:** 10.3390/jcm13185392

**Published:** 2024-09-12

**Authors:** Valentín Freijo, Claudia Navarro, Jordi Villalba

**Affiliations:** 1 Department of Physical Medicine and Rehabilitation, Parc Taulí Hospital Universitari Institut d’Investigació i Innovació Parc Taulí I3PT, Parc Taulí, 1, 08208 Sabadell, Spain; vfreijo@tauli.cat (V.F.); cnavarroo@tauli.cat (C.N.); 2 Service of Orthopaedics and Traumatology, Consorci Hospitalari de Vic, Carrer de Francesc Pla el Vigatà, 1, 08500 Vic, Spain

**Keywords:** knee osteoarthritis, gait, quality of life, knee function

## Abstract

**Background/Objectives**: Treatment of advanced knee osteoarthritis with total knee arthroplasty typically results in an improvement in function, gait, and quality of life, which tend to be affected by the condition. It is, however, necessary to determine the baseline factors that could influence the patients’ postoperative outcome. **Methods**: This is a single-center prospective observational study of patients with advanced knee osteoarthritis (Kellgren–Lawrence grade 3 or 4) treated with total knee arthroplasty. Gait parameters were recorded at baseline and at various postoperative time points using a wireless device. Progression of function was assessed using the Knee Society Score questionnaire and quality of life by means of the EQ-5D and Knee Injury and Osteoarthritis Outcome Score questionnaires. Progression of gait and quality of life was analyzed in all patients, distinguishing between those where baseline velocity was < 1 m/s and those where it was ≥1 m/s. The potential correlation between baseline and postoperative parameters was also evaluated. **Results**: All 119 patients showed a significant improvement in their gait, function, and quality of life parameters at one year from the procedure (*p* < 0.05). No statistically significant differences were found in any of the postoperative subscales, regardless of baseline velocity (< o ≥ 1 m/s) or between any of the baseline or postoperative parameters (r < 0.29). **Conclusions**: Baseline gait parameters in patients with advanced knee osteoarthritis do not appear to bear a statistically significant relationship with function or quality of life outcomes following total knee arthroplasty. Such parameters exhibit a significant improvement one year after surgery.

## 1. Introduction

Knee osteoarthritis is a disabling chronic condition that is highly prevalent the world over, particularly among the elderly. A combination of factors, including cartilage degradation, inflammation, and changes in the underlying bone structure around the joint, contribute to pain. This pain is usually the first symptom experienced by patients, which negatively impacts their function and quality of life (QoL). At advanced stages, treatment usually consists of total knee arthroplasty (TKA), which has proven to be an effective solution that offers significant symptom relief but seldom improves function or QoL in line with patient expectations [1,2,3,4].

Given its quantifiable nature, gait is employed, in conjunction with the well-known patient-reported outcome measures (PROMs), as a tool to evaluate walking function [5]. While PROMs provide the patient’s perception of their condition through different dimensions in standardized questionnaires, gait analysis complements this by offering objective and precise measurements, allowing for a comprehensive view of the patient’s status. Knee osteoarthritis typically causes alterations in patients’ gait parameters, such as slower velocity and a shorter stride length. A preoperative subanalysis carried out as part of this study showed that our patients were no exception, exhibiting velocity measurements below the threshold regarded as a clinical marker for healthy aging (1 m/s) [6]. Moreover, gait parameters were found not to be correlated with knee function and to bear a low correlation with QoL [7,8].

Over the last few years, there has been a proliferation of studies looking at the progression of joint kinematics following TKA. Whether patients experience a postoperative improvement in gait parameters remains controversial, with many instances of partial improvements and indeterminate outcomes having been reported. On the other hand, there is a growing interest in determining all kinds of baseline predictive factors for clinical outcomes of TKA beyond the impact of the pathology on the joint, such as mental and emotional state, opioid use, or weight gain in adulthood, among others [9,10]. Some authors have suggested that preoperative gait parameters could be predictive of postoperative function and QoL outcomes, while others have found a lack of correlation between gait parameters and function and/or QoL. Identifying factors capable of influencing the clinical outcomes of TKA would be extremely beneficial as it would allow for a continuous improvement in therapeutic strategies [11,12,13,14,15].

The purpose of this study was to analyze gait parameters, knee function, and QoL in patients with advanced knee osteoarthritis one year after TKA, as well as the potential influence of baseline gait parameters on postoperative outcomes. Our hypothesis was that baseline gait parameters were correlated with postoperative knee function and QoL.

## 2. Materials and Methods

This is a single-center prospective observational study of a group of consecutive patients with knee osteoarthritis who underwent TKA at the Parc Taulí Hospital between 2020 and 2021. The study was approved by the hospital’s Ethics Committee (code 2020/539, approval date: 18 March 2020). All patients provided their written informed consent.

All patients in the study had been diagnosed with advanced tricompartmental knee osteoarthritis (Kellgren–Lawrence grades 3 or 4) [16] and treated with a TKA. To be included, they had to be able to perform walking tests without using orthopedic aids. Patients who had undergone revision surgery or had diagnosed pathologies that caused additional gait impairment or asymmetries (such as a previous disabling stroke, lumbar stenosis or other neurological, spinal, or musculoskeletal conditions) were excluded from the study.

The patients’ demographic information (sex, age, BMI) was collected in the course of their routine preoperative consultations at the hospital. Those consultations were also used to obtain clinical information about the patients that could prove relevant for the study, e.g., comorbidities, using the Charlson Comorbidity Index, laterality of the condition, and the degree of knee involvement, using the Charnley classification [17]. In addition, an analysis of the patients’ baseline gait, function, and QoL parameters was performed.

Gait was analyzed using the BTS G-Walk system (BTS Bioengineering Corp., Quincy, MA, USA), which consists of a small wireless device that measures various gait parameters using a validated methodology [7,18]. Before each use, the system automatically performs isoinertial calibration of the sensor. For the walking test, patients had to walk at a comfortable speed along an obstacle-free 30 m long walkway wearing flat footwear and without any kind of orthopedic aid. After receiving instructions by the medical team, they were allowed to perform a trial before the measurement to familiarize themselves with the test. The device, attached to the patients’ sacral area (S1), recorded the following gait parameters: velocity (m/s), cadence (steps/min), stride length (m), and propulsion (m/s^2^) with both legs.

Knee function was evaluated using the Knee Society Score (KSS) questionnaire (<60: poor, 60–69: fair, 70–79: good, 80–100: excellent) [19]. QoL was evaluated using the generic EQ-5D-5L questionnaire and the Knee Injury and Osteoarthritis Outcome Score (KOOS), specific for patients with knee osteoarthritis [20,21]. In all three cases, the validated Spanish versions of the questionnaires were used, and the medical team was responsible for administering and recording the patients’ responses.

Following our routinely clinical protocols, patients were followed up at 3, 6, and 12 months postoperatively. Gait parameters were recorded at each appointment to assess any subtle changes that may occur over the short periods of time between consultations. Meanwhile, postoperative knee function and QoL were evaluated at the last follow-up visit.

### Statistical Analysis

All statistical analyses were carried out using the IBM SPSS Statistics for Windows software package (IBM Corp., 2017, Version 25.0, Armonk, NY, USA). A descriptive statistical analysis was performed of all the data collected. The progression of all kinematic gait parameters and of the patients’ scores on the knee function and QoL scales was evaluated at the different follow-up consultations. Patients were classified into two groups according to whether their baseline velocity was < or ≥1 m/s. The progression of velocity over time was analyzed in both groups as well as the patients’ baseline and postoperative knee function and QoL scores and their progression over time. The Shapiro–Wilk test was used to determine the normality of the subsets, and Student’s two-tailed paired test and a chi-squared test were used to confirm or refute the hypotheses generated. Pearson’s correlation coefficient was used to evaluate the correlation of the baseline kinematic gait parameters with the different function and QoL subscales at 12 months postoperatively. Hinkle et al.’s range-based classification system was employed to determine correlations based on Pearson’s coefficient [22]. Statistical significance was set at a value of *p* < 0.05 in all cases.

## 3. Results

**Figure 1:**
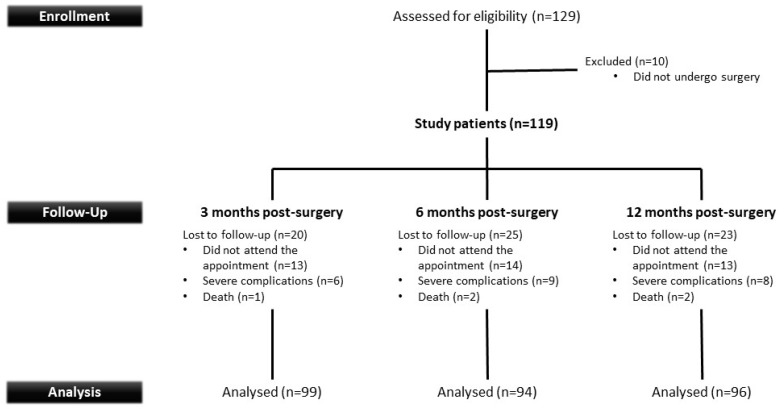
Overall characteristics of the patients included in the study. A total of 10 of the 129 patients identified during the preoperative period were excluded as they were in the end not subjected to the surgical procedure. Some patients were lost to follow-up as they did not show up for their follow-up visits, presented with severe complications, or passed away. However, two patients who missed their 6-month follow-up did attend the final follow-up visit.

The overall characteristics of the patients included in the study are shown in Figure 1. Of the 119 patients who participated in the study, 42 (35.3%) were male and 77 (64.7%) were female. Most patients were obese (BMI > 30 kg/m^2^). The most usual comorbidities recorded were diabetes mellitus (24.4%), cancer (9.2%), cerebrovascular disease (5.0%), and congestive heart failure (5.0%). All the patients’ demographic and clinical data are provided in Table 1.

Preoperatively, 68 (57.1%) patients were found to be below the clinical threshold for healthy aging (velocity < 1 m/s). All spatio-temporal gait parameters showed an improvement at each postoperative follow-up examination. The improvement with respect to the preoperative status was statistically significant at one year after the procedure (Table 2).

**Figure 2:**
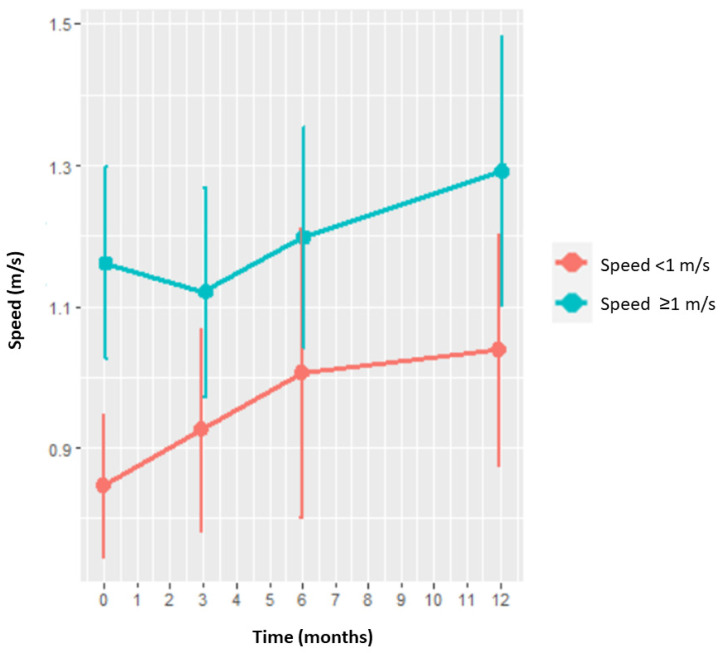
Velocity increase from pre-op to one year after surgery. Patients with a baseline velocity < 1 m/s are shown in orange, while those with a baseline velocity ≥ 1 m/s are shown in blue.

A total of 83.6% of patients with a baseline velocity < 1 m/s and 48.8% of patients with a baseline velocity ≥ 1 m/s achieved a clinically significant velocity improvement (≥0.08 m/s) one year after the procedure. The difference between both groups was statistically significant (*p* < 0.001). The increase in velocity observed at one year from the procedure was significantly higher (*p* = 0.04) in patients with a baseline velocity < 1 m/s, where mean velocity increased by 0.19 ± 0.15 m/s than in patients with baseline velocity ≥ 1 m/s, where the increase was of 0.12 ± 0.21 m/s (Figure 2).

Knee function exhibited a significant improvement during the first year postoperatively (*p* < 0.0001), improving from a poor functional status (<60 on the KSS objective and function scores) to a good functional status on the KSS objective score and an excellent functional status on the KSS function score. Similarly, statistically significant increases (*p* < 0.0001) were observed across all the analyzed QoL subscales during the first year postoperatively, with the greatest improvements being recorded in the realms of activities of daily living (ADLs) and pain, as measured by the KOOS scale (Table 3).

The progression achieved by patients in both baseline velocity groups on the knee function and QoL subscales from pre-op to one year after surgery is shown in Table 4. Although patients with a baseline velocity < 1 m/s obtained lower scores across all preoperative scales, statistically significant differences were only found on the KOOS ADLs (*p* < 0.002) and overall (*p* < 0.020) subscales in patients with a baseline velocity ≥ 1 m/s. No statistically significant differences were found in any of the postoperative subscales. In regard to progression from pre-op to one year after surgery, the only differences were observed in the KOOS symptoms subscale, which showed that the slower group achieved a faster improvement than the faster group (*p* = 0.011).

No correlation was found in our study between the patients’ baseline gait parameters and the function or QoL subscales (r < 0.29) (Table 5).

## 4. Discussion

This study proposes a combined strategy for the evaluation of function and QoL in patients with advanced knee osteoarthritis treated by means of TKA. The strategy consists of the administration of validated questionnaires and the performance of a quantitative gait analysis and involves an evaluation of the patients’ progression, of the relationship between the outcome achieved and baseline velocity, and of the correlation of baseline gait parameters with functional and QoL outcomes.

After any orthopedic procedure, it is the function-related parameters that dictate whether patients will be able to resume normal activity levels and perform their ADLs. Although TKA is a well-established treatment that typically results in a significant clinical improvement, postoperative function levels tend to pale in comparison with those of healthy patients of the same age. The gold standard for evaluating the said parameters are the so-called PROMs, often in the form of self-administered questionnaires. Although validated, practical, and easily used to make comparisons, such instruments have been criticized for their lack of objective measurements and the unquestionable bias that results from the subjectivity of patients’ responses [11,14,23,24].

On the other hand, gait, which can be described in terms of a series of kinematic parameters, is considered to be an indirect tool for quantifying physical activity and is commonly used as a reference when analyzing a patient’s situation before and after an orthopedic procedure. Specifically, patients with advanced knee osteoarthritis often unconsciously adapt their gait trying to avoid placing stress on the affected limb. Such adaptations more often than not have an impact on velocity and cadence, among other gait parameters. Several authors have reported that, although gait kinematics may improve following TKA, this can under no circumstances be taken for granted and, one year following the procedure, there are usually significant differences between the gait patterns and weight-bearing ability of patients who underwent the procedure and healthy individuals [7,11,14,15,23,24].

Our study found a significantly favorable progression of all gait parameters one year from TKA. In regard to velocity, which is the most commonly used indicator to determine the overall evolution of patients over time, our patients were able to walk at a rate of 1.15 ± 0.22 m/s at the end of the follow-up. This was within the ranges reported by several case series with the same follow-up period, which stood between 1.00 ± 0.12 m/s and 1.30 ± 0.10 m/s. As our study did not include a control group made up of healthy individuals, we were not able to determine whether our patients succeeded in normalizing their gait patterns. In this regard, some authors have argued that operated patients are able to achieve a maximum velocity equivalent to 85% of that of healthy individuals [13,15,25,26,27].

In patients with chronic conditions such as knee osteoarthritis, it is particularly difficult to determine how much gait parameters must improve to result in a significant clinical impact. In this study, we have used the velocity threshold of 0.08 m/s, established by Kwon et al. (2009) [28], which is within the 0.07–0.12 m/s range reported in a more recent article by Abigail et al. (2022) [29]. Our patients exceeded a 0.08 m/s improvement at six months from TKA, achieving a statistically significant progression of 0.17 m/s at one year, which also coincided with significant improvements in function and QoL.

Several authors have quantified the progression of function in patients with knee osteoarthritis treated by means of TKA using the KSS. Specifically, the improvements reported stand between 32 and 48 points on the KSS objective subscale and between 18 and 34 points on the KSS function subscale at one year [30,31,32]. The improvement of 33 points observed in our patients on the KSS function subscale is in line with the findings in the literature, whereas the improvement of only 26 points on the KSS objective subscale falls short of such findings. Even so, our patients did progress from a poor to an excellent functional status on both KSS subscales.

As far as overall QoL is concerned, our patients experienced a significant QoL improvement across all EQ-5D and KOOS subscales one year from the procedure. Connelly et al. (2019) [33] established a so-called patient-acceptable symptom state threshold to be determined one year postoperatively. Our patients exceeded this threshold on the majority of subscales employed. Nevertheless, scores on the KOOS ADLs and QoL subscales were slightly lower that Connely’s threshold (82.62 vs. 83.0 points and 65.12 vs. 66.0 points, respectively), and the VAS scale in the EQ-5D questionnaire was a striking 16 points lower than the value regarded as acceptable (67.12 vs. 83.0).

Of all the parameters indicating a patient’s overall health status, a baseline velocity above 1 m/s has been associated with healthy aging. However, to the best of our knowledge, no study has, to date, looked into the relationship between a baseline speed above 1 m/s and the achievement of higher levels of function and QoL following TKA. One year after the procedure, patients with a baseline velocity < 1 m/s exhibited a significantly higher increase in velocity, most of them reaching a clinically significant improvement. These patients also exhibited a greater overall improvement in function and QoL, which was nonetheless only statistically significant for the KOOS symptoms subscale. The final postoperative status was equivalent in both groups across all the subscales analyzed, which suggests that having a baseline velocity above or below the 1 m/s threshold does not have any impact on the patients’ postoperative functional QoL or outcomes, and slower patients typically have a greater degree of improvement across the analyzed parameters [6,28].

No correlation was found at one year from TKA between the patients’ postoperative gait parameters and their function and QoL, which suggests that their baseline gait did not exert a marked influence on the results of the treatment. Using a less conservative Pearson coefficient threshold such as those employed by other authors, these absences of correlation would be regarded as low positive correlations. In spite of this, we have decided to stick to our more conservative approach as it is the one validated in the statistical literature [7].

Our results are in line with the findings of two studies that observed a lack of correlation between baseline velocity and the patients’ postoperative scores on the KOOS subscales and the Lawton–Brody Instrumental Activities of Daily Living Scale, both widely used instruments for the evaluation of QoL [12,34]. Similarly, Yocum et al. (2023) analyzed the correlation between several gait parameters and QoL and physical status and only found a low correlation between the number of steps taken daily and the physical subscale of the Veterans RAND 12-Item Health Survey [13]. Our hypothesis was based on the empirical observations we have gained from years of experience treating these patients. Nevertheless, as previous studies have pointed out, it is undeniable that the significant improvement in the lives of many patients following TKA might explain why, despite starting the study with poor gait parameters, their function and QoL have improved so markedly that no correlations with their preoperative status are found. If anything, this only highlights the effectiveness of the treatment.

However, the influence of baseline gait parameters on the clinical outcomes following TKA cannot be ruled out entirely. Kluge et al. (2018) [11] reported that their patients’ stride time and stride length were able to predict postoperative function at one year in up to 89% of cases, although those of their patients with more favorable gait parameters experienced a worsening of function during the postoperative period, which is in line with the findings of Berliner et al. (2017) [9], whose patients with better preoperative function were less likely to experience a significant improvement after the procedure.

We believe that our study could be helpful for clinicians in terms of exploring new approaches to other factors influencing clinical outcomes, re-evaluating rehabilitation protocols, or managing the expectations of patients who perceive themselves as slower and tend to have a pessimistic view of their postoperative results. But, as ever, more research is needed using a larger number of variables to better understand the influence of the various baseline factors on clinical outcomes. This would help anticipate patient progression and tailor our therapeutic strategies accordingly.

Our study has several limitations. When performing the data analysis, we did not apply non-dimensional normalization based on patients’ somatometric characteristics. We acknowledge that this normalization is crucial in many gait studies, particularly those involving children or diverse populations with varying ages, heights, and levels of physical activity [35]. However, our sample is rather more homogeneous, consisting of elderly patients with advanced-stage knee osteoarthritis. Although this statistical approach has been used in previous studies with similar patient cohorts [14,27], it is not as common as in other fields of gait analysis. Nevertheless, the main limitation of this study lies in the fact that the evaluation of gait was performed using a simple 30 m walking test, chosen due to its simplicity and ease of administration in the clinical setting. Future studies could address this limitation by expanding gait testing to real-life activities outside of a clinical setting. With the advent of wireless gait analysis systems, patients could be remotely monitored at home while performing routine activities, such as climbing and descending stairs. This approach would help identify the actual difficulties they encounter in their ADLs through quantifiable remote monitoring techniques.

In conclusion, baseline gait parameters in patients with advanced knee osteoarthritis do not seem to bear a statistically significant relationship with the outcomes of TKA in terms of function or QoL.

## Figures and Tables

**Table 1:** Baseline demographic and personal characteristics of the patients in the study. BMI: Body Mass Index.

		N = 119
Age (years)		69.4 ± 7.9
BMI (kg/m^2^)		33.8 ± 5.9
Charlson Comorbidity Index		
	Severe	5 (4.2%)
	Mild	13 (10.9%)
	No comorbidities	101 (84.9%)
Affected knee		
	Left	62 (52.1%)
	Right	57 (47.9%)
Charnley classification		
	A	84 (70.6%)
	B1	33 (27.7%)
	C1	1 (0.8%)
	C3	1 (0.8%)

Charnley classification. A: unilateral knee osteoarthritis; B1: unilateral total knee arthroplasty, opposite knee arthritic; C1: total knee replacement, but remote arthritis affecting ambulation; C3: unilateral or bilateral total knee arthroplasty or bilateral total hip replacement.

**Table 2:** Progression of gait parameters over time. The *p*-value is shown for the comparisons of each preoperative variable and at 12 months postoperatively.

Time	Baseline	3 Months	6 Months	12 Months	*p*-Value
Speed (m/s)	0.98 ± 0.20	1.01 ± 0.17	1.09 ± 0.21	1.15 ± 0.22	<0.001
Cadence (steps/min)	105.7 ± 10.1	106.1 ± 9.1	110.9 ± 10.2	115.9 ± 10.8	<0.001
Left stride length (m)	1.12 ± 0.18	1.15 ± 0.17	1.17 ± 0.17	1.19 ± 0.18	<0.001
Right stride length (m)	1.12 ± 0.18	1.15 ± 0.17	1.17 ± 0.17	1.19 ± 0.18	<0.001
Left propulsion (m/s^2^)	5.35 ± 1.92	5.48 ± 1.43	6.14 ± 1.93	6.64 ± 1.97	<0.001
Right propulsion (m/s^2^)	5.43 ± 2.02	5.57 ± 1.74	6.32 ± 2.13	6.79 ± 2.36	<0.001

**Table 3:** Evaluation of knee function and QoL at pre-op and at one year from surgery.

Questionnaires		N	Baseline	12 Months	*p*-Value
**KSS**					
	Objective	94	53.13 ± 11.63	79.04 ± 9.52	<0.0001
	Function	94	55.47 ± 21.31	88.13 ± 11.77	
**EQ-5D**					
	Overall	92	0.43 ± 0.25	0.85 ± 0.19	<0.0001
	VAS	92	59.07 ± 17.78	67.12 ± 17.34	
**KOOS**					
	Symptoms	91	47.98 ± 20.37	83.3 ± 14.76	<0.0001
	Pain	89	37.45 ± 18.91	85.75 ± 18.16	
	ADLs	90	33.76 ± 19.42	82.62 ± 18.64	
	Sport/recreation function	89	4.49 ± 9.26	39.88 ± 19.90	
	QoL	90	24.39 ± 17.03	65.12 ± 26.23	
	Overall	91	29.74 ± 13.95	71.38 ± 16.88	

**Table 4:** Progression of knee function and QoL from pre-op to one year after surgery in each group analyzed. An asterisk (*) indicates that the result is statistically significant (*p* < 0.05)

Scales		Time	Velocity < 1 m/s	Velocity ≥ 1 m/s	*p*-Value
KSS	Objective	Baseline	51.51 ± 12.50	52.90 ± 11.50	0.542
		12 months	78.93 ± 9.91	78.66 ± 9.19	0.892
		Difference	26.50 ± 15.39	25.10 ± 15.36	0.664
	Function	Baseline	52.76 ± 21.75	56.84 ± 22.97	0.332
		12 months	87.00 ± 13.11	89.02 ± 9.89	0.410
		Difference	32.13 ± 28.86	33.00 ± 28.73	0.880
EQ-5D	Overall	Baseline	0.41 ± 0.25	0.47 ± 0.24	0.200
		12 months	0.77 ± 0.23	0.78 ± 0.23	0.753
		Difference	0.41 ± 0.25	0.30 ± 0.24	0.104
	VAS	Baseline	54.70 ± 17.80	61.46 ± 19.24	0.055
		12 months	66.18 ± 17.64	67.56 ± 17.61	0.705
		Difference	8.68 ± 18.87	7.18 ± 21.30	0.722
KOOS	Symptoms	Baseline	44.55 ± 19.51	51.45 ± 20.84	0.070
		12 months	85.78 ± 12.72	80.51 ± 16.46	0.080
		Difference	41.68 ± 23.44	29.21 ± 21.96	0.011 *
	Pain	Baseline	34.55 ± 19.89	39.29 ± 18.37	0.193
		12 months	84.89 ± 18.51	86.49 ± 17.59	0.670
		Difference	49.52 ± 27.19	46.87 ± 22.86	0.620
	ADLs	Baseline	27.95 ± 17.70	39.17 ± 19.19	0.002 *
		12 months	81.58 ± 20.72	82.37 ± 18.49	0.848
		Difference	52.43 ± 25.77	42.62 ± 23.55	0.063
	Sports	Baseline	4.15 ± 14.32	6.20 ± 10.57	0.380
		12 months	39.82 ± 19.72	38.78 ± 20.58	0.803
		Difference	34.72 ± 27.69	33.12 ± 17.68	0.737
	QoL	Baseline	21.38 ± 17.04	26.16 ± 16.79	0.137
		12 months	65.13 ± 27.15	64.73 ± 25.39	0.942
		Difference	42.43 ± 30.83	38.53 ± 26.97	0.526
	Overall	Baseline	26.49 ± 13.06	32.42 ± 13.65	0.020 *
		12 months	71.49 ± 17.07	70.59 ± 16.84	0.796
		Difference	44.21 ± 22.13	38.10 ± 18.21	0.159

**Table 5:** Correlation (r) between baseline gait parameters and function and QoL outcomes. An asterisk (*) indicates that the result is statistically significant (*p* < 0.05). All values indicate an absence of correlation (r < 0.29).

Gait Parameters	KSS		KOOS						EQ-5D	
	Objective	Function	Symptoms	Pain	ADLs	Sport/Recreation Function	QoL	Overall	Overall	VAS
Velocity	0.04	0.14	0.17	0.08	0.14	0.11	0.01	0.01	0.05	0.05
Cadence	0.17	0.05	0.20	0.08	0.05	0.17	0.17	0.16	0.12	0.02
Left stride length	0.13	0.17	0.09	0.13	0.15	0.03	0.10	0.05	0.11	0.17
Right stride length	0.13	0.17	0.09	0.13	0.15	0.03	0.10	0.05	0.11	0.18
Left propulsion	0.10	0.20	0.11	0.06	0.16 *	0.01	0.08	0.05	0.16	0.17 *
Right propulsion	0.10	0.20	0.13	0.05	0.15	0.11	0.01	0.01	0.15	0.16 *

## Data Availability

The datasets used and/or analyzed during the current study are available from the corresponding author on reasonable request.
